# The impact of employees' pro-environmental behaviors on corporate green innovation performance: The mediating effect of green organizational identity

**DOI:** 10.3389/fpsyg.2022.984856

**Published:** 2022-11-08

**Authors:** Zujie Cheng, Banggang Wu, Xiaoyu Deng, Wei Li

**Affiliations:** ^1^Wuliangye Yibin Co., Ltd., Yibin, China; ^2^Business School, Sichuan University, Chengdu, China; ^3^Business School, Beijing Technology and Business University, Beijing, China

**Keywords:** green innovation performance, green organizational identity, innovation resistance, employees' pro-environmental behaviors, leader's pro-environmental behaviors

## Abstract

Employees' behaviors, as well as the employees' pro-environmental behaviors (PEB), affect the company in many dimensions. Although green innovation performance (GIP) has become an important measurement of a corporate's green development, research investigating PEB from the employees' perspective remains scarce, especially in emerging markets. Therefore, in this study, we developed an original framework to explore the effects of employees' PEB on corporate GIP and examined the underlying mechanism by conducting a survey in China. The results of the empirical analysis showed that employees' PEB increases corporate GIP by positively influencing green organizational identity (GOI). In addition, we also proved how leaders' PEB positively influences GIP, whereas innovation resistance (both technology resistance and resource resistance) has a negative effect on GIP. This study attempted to contribute to theoretical research and practical decision-making in the field of green organizational behavior.

## Introduction

To provide aid to the global concern for environmental issues, such as carbon emissions, companies must engage in the green economy by adapting to external policy requirements and achieving sustainable development (Samad et al., 2021). Corporate green innovation, once considered an unnecessary investment in corporate performance, has become important in improving the ability of companies to achieve sustainability and gain a unique competitive advantage, especially in emerging markets (Kong et al., 2021). For example, the Chinese government has included green innovation as a company's key performance indicators and requires state-owned manufacturing companies to participate in green technology innovation projects. However, studies on this topic are scarce. Most of the existing studies found that macro factors or external factors, such as policy instruments (Stucki et al., 2018), limited company resources (Hiz et al., 2018), and financing constraints (Yu et al., 2021), could affect GIP. Although some studies examined internal corporate processes such as management practices (Samad et al., 2021) or transformational leadership (Singh et al., 2020), few studies considered the impact of employees' individual behavior on the green economy, which is measured as employees' PEBs in most cases (Boiral et al., 2015). Employees' PEB plays an essential corporate role in purchasing (Arvola et al., 2008), reducing consumption (Iyer and Muncy, 2009), green travel (Carrus et al., 2008), and recycling (Boiral et al., 2015). Numerous studies explored the factors that can increase employees' PEB (Robertson and Barling, 2013; Afsar et al., 2016; Kim et al., 2016; Yuriev et al., 2020; Shah et al., 2021), while studies hardly tested how employees' PEB affects corporate performance. In any event, we must fully understand the relationship between employees' PEB and corporate GIP.

Furthermore, although individual employees do not directly contribute to corporate performance in a significant way, especially in emerging markets, their organizational identity affects organizational behavior, according to the organizational identity theory (Gioia, 1998). Correspondingly, GOI can connect employees' PEB to corporate GIP by enhancing employees' green personal–social identification (Chen, 2011). Prior studies discussed the impact of GOI on GIP from the perspectives of corporate social responsibility and green innovation strategy (Song and Yu, 2018), environmental organizational legitimacy (Soewarno et al., 2019), green organizational climate (Zafar et al., 2022), and environmental leadership (Robertson and Carleton, 2018). However, research is scarce on the concept of the employee as a fundamental component of a company. Therefore, we aimed to expand the current literature by exploring the mediating effect of GOI on the relationship between employees' PEB and corporate GIP.

Current studies showed that several factors affect GIP, including external barriers to the implementation of environmental strategies and internal management practices of companies (Samad et al., 2021). The shift to green innovation requires resources and capabilities, making innovation resistance an important issue (Abdullah et al., 2016). For example, Murillo-Luna et al. (2008) pointed out that a lack of resources hinders the implementation of corporate environmental strategies, limits technology development, and thus reduces green innovation performance. Innovation resistance commonly refers to technology resistance and resource resistance (Gohoungodji et al., 2020). However, as there is a lack of research connecting innovation resistance to micro-level employees' PEB and macro-level corporate GIP, we attempted to fill the research gap through this study. Still, most of the studies discussed that the leaders' PEB may affect employees' PEB while ignoring the other mechanism. To this end, the moderating effect of innovation resistance and leaders' PEB should be further explored in our framework.

In the context of the green economy, in this study, we focused on corporate GIP from the employees' perspective, which plays an important role in corporate performance but has not been fully taken into account as a dependent variable, attempted to determine the impact of employees' PEB on GIP. Moreover, to find the mechanism, we also tested the mediating effect of GOI and the moderating effect of leaders' PEB and innovation resistance. The results of this study indicate that employees' PEB positively affects corporate GIP through GOI, and leaders' PEB has a positive moderating effect, whereas innovation resistance has a negative moderating effect, on the relationship between employees' PEB and corporate GIP.

This study contributes to the existing literature by considering employees' PEB, an internal and individual-level variable, as an antecedent factor of corporate GIP, and in doing so, we will enhance the existing research that mainly focuses on external policy dimensions or internal management dimensions (Kong et al., 2021). Furthermore, we will refer to the mediating effect of GOI, which enhances the literature by testing a new perspective that links the employee and the company in the green economy context (Zafar et al., 2022). Finally, we took into account both an external factor (innovation resistance) and an internal factor (leaders' PEB) as moderators in our framework, expanding the current research framework in this field. In this study, we also aimed to improve GIP from a managerial perspective.

## Theoretical background and research hypothesis

### Corporate green innovation performance

From the natural resource-based view, the natural environment can limit the sustainable competitive advantage of a company (Hart, 1995). Green innovation is an important tool for companies to balance economic development and environmental management (Afsar et al., 2016). Green innovation refers to new goods, services, processes, or management systems that can achieve energy-saving and environmental protection, which compensates for the environmental management costs invested by the company (Saunila et al., 2018; Stucki et al., 2018).

There are abundant studies exploring the driving factors of GIP from a variety of dimensions, including both external factors and internal drivers (Samad et al., 2021). From the external view, most studies focused on political and economic factors. For example, Stucki et al. (2018) found that encouraging policy instruments can increase GIP by creating a supportive law or policy system. The current literature also pointed out some factors that may block green innovation, such as limited company resources (Hiz et al., 2018) and financing constraints (Yu et al., 2021). From the internal view, on the contrary, the existing research discussed the impact of management practices (Samad et al., 2021) or transformational leadership (Singh et al., 2020) on GIP from a managerial perspective. For example, Samad et al. (2021) tested whether human resource revolution in green management would increase green innovation. Still, however, these internal factors did not refer to the employees' behavior. Since employees are the most fundamental component of a company, the influence of their behavior is worth exploring in the issue of green innovation.

### Employees' pro-environmental behaviors

Employees' PEB, defined as the “willingness to engage in pro-environmental activities”, achieve many concerns in the past few decades (Scherbaum et al., 2008). Most of the studies focused on the factors that could affect employees' PEB, including workplace spirituality, intrinsic motivation, environmental passion, the CSR strategy of the organization, and leadership (Robertson and Barling, 2013; Afsar et al., 2016; Kim et al., 2016; Yuriev et al., 2020; Shah et al., 2021).

The existing literature supported that employees' behavior has a significant impact on the organization, while few of those literature focused on the employees' PEB (Iyer and Muncy, 2009; Shah et al., 2021). It has been found that employees' PEB can lead to a “win–win” situation: not only does it protect the natural environment but also improves environmental performance, leader effectiveness, and employee job satisfaction (Robertson and Barling, 2013). However, the specific impact of employees' PEB on corporate performance still needs to be explored. Based on the previous studies on the relationship between employees' behaviors and organizational performance, we can infer that employees' PEB, a type of employees' behaviors, can also impact the corporate. Furthermore, positive employees' PEB may increase green innovation in a company.

Therefore, this study proposes the following assumption:

*Hypothesis 1: Employees' PEB positively influences corporate GIP*.

### The mediating effect of green organizational identity

From the perspective of the organization identity theory, an individual's organizational identity and emotional base can influence organizational behavior, while the identity can influence employees' goal-seeking persistence (Gioia, 1998). Organizational identity can be formed when organizational identity and organizational members' self-concept coincide (Dutton et al., 1994). When an employee shows PEB in a company, which encourages environment-friendly spirit in the workplace, organizational identity between the employee and the corporate would be tighter than that for an employee who does not show PEB (Gohoungodji et al., 2020). Hence, we made the following assumptions:

*Hypothesis 2: Employees' PEB positively influences GOI*.

GOI refers to a jointly constructed organizational identity on environmental management and a protection organization identity model (Chen, 2011). GOI identifies the cognitive structure that legitimizes environmental management as an organizational identity structure (Soewarno et al., 2019). This common cognitive structure can enhance the value and sense of belonging of the organization members so that members of the company are motivated by the unified identity of the organization and deepen the understanding of the corporate environmental strategy, which finally contributes to the corporate performance (Robertson and Carleton, 2018). If environmental issues become a mainstream of organizational identity within an organization, they can be interpreted as positive meanings that could encourage the members of the organization to contribute more commitment to environmental activities (Sharma, 2000).

*Hypothesis 3: GOI positively influences corporate GIP*.

### The moderating effect of leaders' pro-environmental behaviors

The natural resource-based view complements the traditional perspective on understanding and application of corporate leadership, considering the leader as a component of a positive element, which indicates that corporate leaders resolve company environment conflicts and problems by identifying environmental issues, developing environmental strategies, and communicating with other helpful organizations (Zafar et al., 2022). Meanwhile, in the social identity theory, organizational identity is formed in the leader's understanding of issues and beliefs that guide and drive organizational behavior (Gioia, 1998). Organizational leaders are ideally placed to serve as role models because of their position, status, and power (Brown et al., 2005). Without senior corporate leaders' support, neither can employees' GOI be achieved nor can green innovation ideas be realized (Abdullah et al., 2016). Thus, a leader who supports environment-friendly behavior would positively contribute to the overall corporate green development.

Therefore, this study proposes the following assumption:

*Hypothesis 4: Leaders' PEB positively moderates the effect of employees' PEB on corporate GIP*.

### The moderating effect of innovation resistance

From the natural resource-based view, some research studies focused on the positive elements that could help increase GIP, while other studies were concerned with the negative elements that may prevent the development of GIP, such as innovation resistance (Heidenreich and Spieth, 2013). As an important external factor, innovation resistance blocks the process of green innovation, for example, Murillo-Luna et al. (2008) pointed out that a lack of resources hinders the implementation of corporate environmental strategies, limits technology development, and thus reduces green innovation performance. Innovation resistance commonly refers to technology resistance and resource resistance (Gohoungodji et al., 2020). van Klyton et al. (2021) explored the technology level in the innovation process, which plays a positive role in final corporate performance, as well as the corporate's initial resources in the innovation process. Similar results can also be found in a recent study (Heidenreich and Spieth, 2013). Therefore, this study proposes the following assumption:

*Hypothesis 5: Innovation resistance negatively moderates the effect of employees' PEB on corporate GIP*.

**Figure 1:**
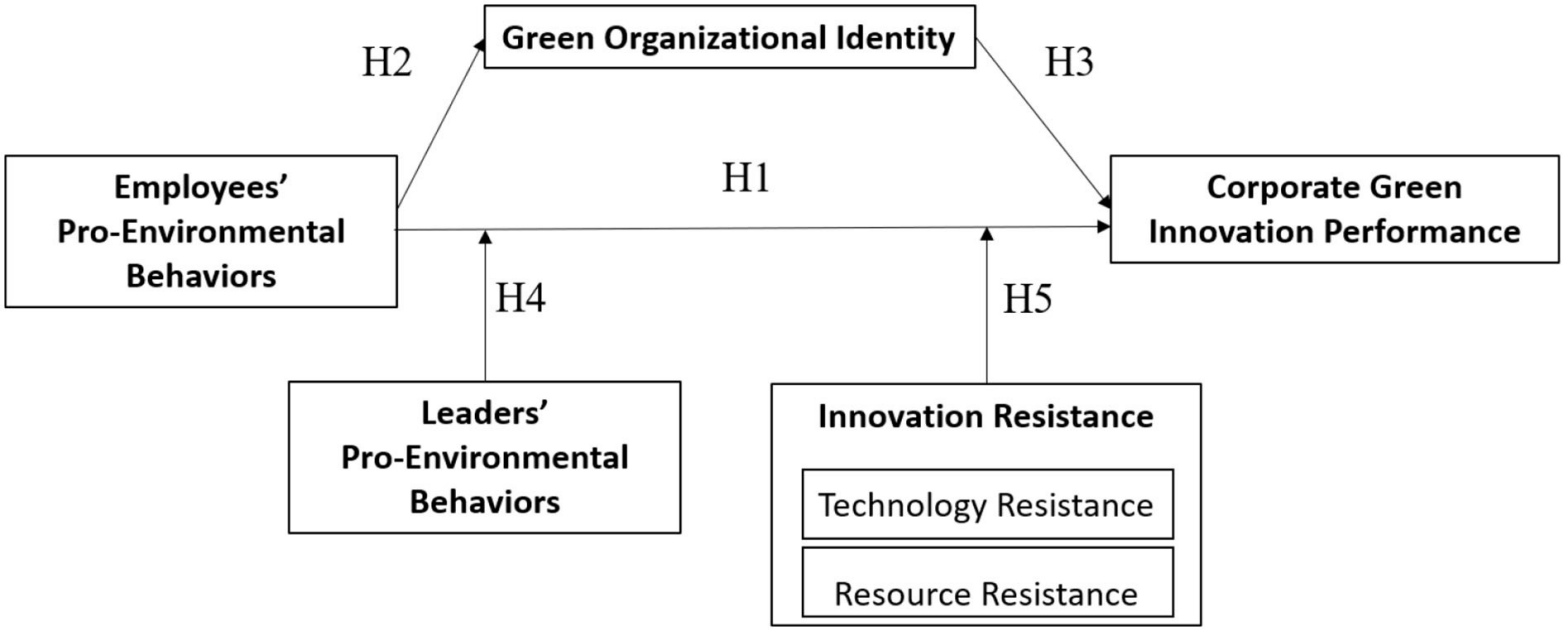
Research framework.

In this section, we built up our research hypotheses based on two mainstream theories: the natural resource-based view and the organization identity theory. We considered the individual-level employees' PEB and organization-level GIP and attempted to discover a relationship between them and the underlying mechanisms. Figure 1 represents the proposed research framework.

## Research design

### Questionnaire design

To test the hypotheses proposed in this study, we designed a questionnaire survey and then collected the data through that the survey.

In the questionnaire design process, four stages were involved: First, we chose the research constructs and measurement items of all the key variables mentioned in the previous research framework mentioned in this study. The measurement scale for each variable was based on the existing literature, referring to employees' PEBs (Robertson and Barling, 2013), corporate GIP (Chen et al., 2006), GOI (Gioia and Thomas, 1996), leaders' PEBs (Robertson and Carleton, 2018), and innovation resistance (Delgado-Ceballos et al., 2012). Second, as the participants were from China and the original scales of the questionnaire was in English, we translated them into Chinese and sent them to many professional researchers to ensure translation accuracy. Third, before the formal survey, we sent the pre-survey questionnaires to 50 participants, including some experts in the organization behavior field and then modified the questionnaire according to their comments and the pre-survey results on the questionnaire design. Finally, we uploaded the modified questionnaire to the online data collection platforms. The final formal questionnaire contained three parts: The first section details the purpose of the questionnaire, emphasizing that data collection is purely conducted for academic purposes and guarantees the absolute confidentiality of the information. The second section, the core section, collects data on all the variable measurement items we want to explore in this study, including employees' PEBs, corporate GIP, GOI, leaders' PEBs, and innovation resistance. The third part includes demographic information, including gender, education level, and industry. The composition and corresponding items of the questionnaire are provided in column 1 and column 2 in Table 1.

**Table 1:** Research on constructs, measurements, item loadings, and validities.

**Variable**	**Item**	**Standard Loading**	**Cronbach's Alpha**	**CR**	**AVE**
EPEB	(1_1)I print double sided whenever possible	0.801	0.805	0.895	0.630
	(1_2)I put recyclable material in the recycling bins	0.772			
	(1_3)I turn lights off when not in use	0.819			
	(1_4)I take part in environmentally friendly programs	0.783			
	(1_5)I make suggestions about environmentally friendly practices to managers to increase my organization's environmental performance	0.794			
CGIP	(2_1)The company uses the fewest amount of materials to comprise their products for conducting the product development or design	0.813	0.783	0.839	0.635
	(2_2)The manufacturing process of the company effectively reduces the use of raw materials	0.797			
	(2_3)The manufacturing process of the company effectively reduces the consumption of water, electricity, coal, or oil	0.780			
GOI	(3_1)The company's top managers, middle managers, and employees have a sense of pride about the company's environmental goals and missions	0.779	0.790	0.866	0.618
	(3_2)The company's top managers, middle managers, and employees feel that the company has carved out a significant position with respect to environmental management and protection	0.772			
	(3_3)The company's top managers, middle managers, and employees feel that the company have formulated well defined environmental goals and missions	0.753			
	(3_4)The company's top managers, middle managers, and employees identify that the company highly pay attention to environmental management and protection	0.837			
LPEB	(4_1)My leader acts as an environmental role model	0.851	0.792	0.883	0.715
	(4_2)My leader recognizes my ability to improve our organization's environmental performance	0.831			
	(4_3)My leader spends time developing my skills to contribute to our organization's environmental performance	0.854			
IR	(5_1)Lack of financial resources	0.774	0.829	0.893	0.625
	(5_2)Lack of environmental awareness among employees and managers	0.742			
	(5_3)Unfavorable attitudes among workers and directors	0.838			
	(5_4)Inadequate environmental training and expertise among managers	0.808			
	(5_5)Difficulty in overcoming environmentally unfriendly practices	0.789			

### Data collection and samples

We gathered high-quality data from the questionnaire uploaded in a Chinese data market application named Credamo (https://www.credamo.com/#/), which is one of the most famous, commonly used, and professional data collection platforms in China.

The platform has many registers countrywide, and we invited participants from manufacturing organizations to participate in our survey. Previous studies proved that manufacturing is one of the leading causes of environmental degradation, especially in emerging or developing areas (Samad et al., 2021). Therefore, we aimed to identify the employees' PEB in GIP as the mediating and moderating factors.

In this study, we collected 500 valid questionnaires by incentivizing the participants on completing all the questions. Specifically, in Credamo, we can identify whether the participants effectively took part in the survey by identifying the questionnaires that had the same answer to all the questions or those who finished in an unreasonably short time. The 500 participants were all employees in a company, and they were asked to answer the questions from their perspective; for example, they were asked to answer the employees' PEB questions based on their own daily behavior, while they were asked to answer the leaders' PEB questions based on their observations of their leaders in daily work. The samples cover all provinces, cities, and regions in China, with participants of different gender, age, and education. Apart from these constructs, we also considered the control variables in the following analysis and econometric models, such as gender, age, and other demographic variables, which are also consistent with the existing literature, and collected them in the questionnaire, (Robertson and Barling, 2013; Samad et al., 2021). Table 2 presents the sample characteristics statistics.

**Table 2:** Sample characteristics (*N* = 500).

**Characteristics**	**Classification**	**Sample**	**Percentage (%)**
Gender	Men (=0)	198	39.6
	Women (=1)	302	60.4
Age	Youth (18_30 years)	254	50.8
	Middle aged (31_50 years)	193	38.6
	Elderly (51_60 years)	53	10.6
Industry	Manufacture	98	19.6
	Construction	246	49.2
	Real Estate	61	12.2
	Energy	67	13.4
	Other	28	5.6
Education	High School/Technical	14	2.8
	College	57	11.4
	Undergraduates	359	71.8
	Graduate or above	70	14.0

From Table 2, we can understand that, according to the distribution of gender, age, type of manufacturing organizations, and education, the samples collected through Credamo show a qualified generalizability toward and representativeness of the targeted population. In Chinese manufacturing companies, female employees account for more than half of the total number of employee, most of whom work in sales departments or as secretaries, since a decade ago (Otis, 2008), which may lead to a gender imbalance in our samples. In addition, since there is an education threshold, a university degree in most cases, to enter large-scale manufacturing organizations in China (Guan and Frenkel, 2018), undergraduates account for a large percentage of the industry, which is consistent with our sample characteristics.

## Empirical results

### Correlation

In this study, we adopted multiple linear regression for data analysis. Before constructing the empirical model, we derived the correlation matrix to test whether there is multicollinearity in our data. Table 3 shows the result of the major variables in this study. Columns 2 and 3 present the mean and standard deviation of the independent variables and the dependent variables as well as the mediator and moderators.

**Table 3:** Estimation results of SEM.

			**Estimate**	**S.E**.	**C.R**.	**P**
GOI	<–	EMPLOYEES' PEB	0.034	0.005	6.893	0.001
CGIP	<–	GOI	0.572	0.081	7.021	0.001
CGIP	<–	EMPLOYEES' PEB	0.682	0.114	5.820	0.004
CGIP	<–	LPEB	0.539	0.089	6.034	0.002
CGIP	<–	IR	−0.408	0.083	−4.901	0.011
CGIP	<–	EMPLOYEES' PEBxLPEB	0.041	0.008	5.998	0.003
CGIP	<–	EMPLOYEES' PEBxIR	−0.113	0.016	−6.901	0.001
CGIP	<–	Industry	0.030	0.032	0.942	0.603
CGIP	<–	Gender	−0.049	0.012	−4.108	0.032
CGIP	<–	Age	−0.012	0.003	−4.655	0.017
CGIP	<–	Education	0.033	0.006	5.773	0.006

The results showed that all the coefficients are <0.5, indicating that no multicollinearity effects exist in our model. In addition, we also calculated the VIFs for our empirical models, and the results showed that the VIFs of all the empirical model are <10 (Ryan and Frederick, 1997), which excludes potential multicollinearity problems.

The matrix shows that the correlation coefficient between employees' PEB and CGIP is positive, which is consistent with our hypothesis on the main effect. Moreover, the correlation coefficients between GOI and employees' PEB and between GOI and CGIP are both significant, which could support our mediating hypothesis from a model-free perspective. While the correlation coefficient between LPEB/IR and CGIP is smaller than the correlation coefficient between employees' PEB and CGIP, it shows that the moderating effect is worth further exploration using a comprehensive model.

### Reliability and validity analysis

In this study, we first calculated the standard loadings of each question, respectively, to ensure the reliability of the item, and the results given in column 3 of Table 1 show that all the values are >0.7, indicating reliability of the qualified items (Fornell and Larcker, 1981). Then, we tested the reliability of the questionnaire data by calculating the value of Cronbach's α coefficient and composition reliability (CR). The results in column 4 and column 5 of Table 1 show that the values of all questions are >0.7, which indicate acceptable reliability and internal consistency (Hair et al., 2011). Moreover, in this study, we tested the convergence validity by calculating average variance extracted (AVE) values accordingly; as shown in column 6 of Table 1, we can find that all the AVE values are >0.5, which satisfied the standard value (Fornell and Larcker, 1981). The standard loadings, Cronbach's α coefficient, CR, and AVE are all calculated by SPSS 24.0 and AMOS 22.0.

### Model verification

**Figure 2:**
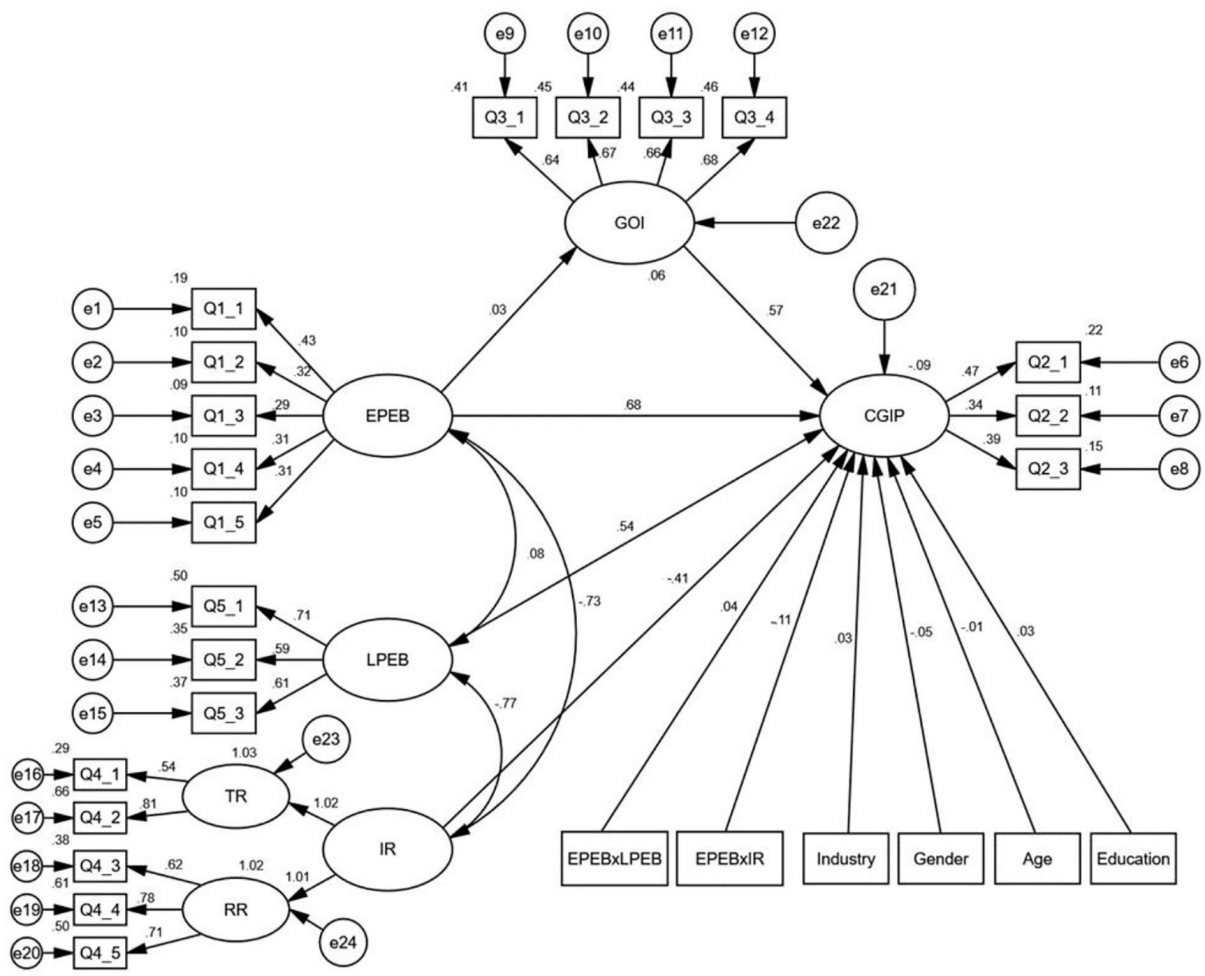
Path of structural equation model.

According to the research framework (Figure 1) and the previous hypothesis, we applied a structural equation model (SEM) to examine the questionnaire survey data. The path of the structural equation model is shown in Figure 2.

We used AMOS 22.0 to carry out the model analysis in this study. We tested the model-fitting indexes, which measured the consistency between hypothesis model and data. The model-fitting indexes (CMID/DF = 1.802, RMSEA = 0.031, GFI = 0.904, AGFI = 0.913, CFI = 0.901) indicated that the fitting degree of the model is acceptable according to the standards (Fornell and Larcker, 1981).

The estimated results of our model are present in Figure 2 and Table 3.

### Main effect

The fundamental research question of this study is to explore the impact of employees' PEB on corporate GIP. The results presented in Table 3 indicate that the effect of employees' PEB on corporate GIP is significantly positive (β = 0.682, *p* < 0.001). Therefore, H1 (employees' PEB positively influences corporate GIP) is supported.

### Moderating effect

From this study, we found two moderators: leaders' PEB and innovation resistance. To test the moderating effect, we added the interaction term between employees' PEB and leaders' PEB (i.e., employees' PEB ×LPEB) and the interaction term between employees' PEB and innovation resistance (i.e., employees' PEB ×IR). Specifically, in the study, we divided innovation resistance into two different dimensions, technology innovation resistance (TR) and resource innovation resistance (RR), as presented in Figure 2. Table 3 presents the results of the moderating effect. The results showed that the impact of employees' PEB ×LPEB on corporate GIP is significantly positive (β = 0.041, *p* < 0.001), indicating that leaders' PEB positively moderates the effect of employees' PEB on GIP. Thus, H4 is supported. The impact of employees' PEB ×IR on CGIP is significantly negative (β = −0.11, *p* < 0.001), indicating that innovation resistance negatively moderates the effect of employees' PEB on GIP. Thus, H5 is supported.

### Mediating effect

We also tested the mediating effect of GOI on the impact of employees' PEB on GIP. We used the bootstrap method to test the mediating effect. The estimated results are presented in Table 4.

**Table 4:** Mediating effect of GOI.

	**Estimate**	***P*-value**	**Bootstrap (95% CI)**
Direct effect	0.049	0.000	[0.147, 0.309]
Indirect effect	−0.184	0.001	[−0.120, −0.064]
Total effect	0.075	0.000	[0.071, 0.136]

From the results, we can infer that the direct, indirect, and total effects are all significant (*p* < 0.001), which satisfy the model tests and support H2 (employees' PEB positively influences GOI) and H3 (GOI positively influences corporate GIP).

## Discussion and conclusion

### Discussion

We explored the impact of employees' PEB on corporate GIP, as well as the mediating effects of GOI. In addition, we also tested the moderating effect of leaders' PEB and innovation resistance (technology innovation resistance and resource innovation resistance). Accordingly, we attained three main conclusions based on our analysis.

First, we found a positive relationship between employees' PEB and GIP, indicating that the higher the employees' PEB, the higher the green innovation performance of the company. Second, we considered the role of GOI, an important element of corporate culture, and indicated that GOI shows a significant mediating effect on the impact of employees' PEB on GIP, showing that employees' PEB affects CGIP by influencing the GOI of the company. Third, we identified the positive moderating effects of leaders' PEB and negative moderating effects of IR on the relationship between employees' PEB and GIP; that is, when the employees' PEB is stable, leaders' PEB would lead to higher GIP, while greater innovation resistance would decrease the GIP. Furthermore, we divided innovation resistance into technology and resource dimensions and then explored them. The results showed that resource innovation resistance has a larger impact on GIP than on technology innovation resistance. All the hypotheses in the framework of this study are supported.

### Theoretical implications

Findings from this research offer important theoretical implications. First, we explored the antecedents of CGIP by incorporating employees' PEB into its analysis, which enriched the existing research that mainly focuses on the firm type (Miller, 1983), green creativity (Chen and Chang, 2013), and human resources (Dumont et al., 2017). We examined the effect from the employees' perspectives, instead of the firm's perspective, which is the focus of existing studies. In the existing studies, firms' perspectives purely emphasize the effect of firms' decisions, ignoring the process of the impact. Our study not only includes the completion of tasks according to employees' PEB but also covers the environment-friendly behaviors produced by employees to achieve sustainable development.

Second, we referred to the mechanism that employees' PEB would affect CGIP through the GOI, which enriched the previous literature by testing a new perspective from the company culture dimension that is rarely considered in the previous studies as a mediator (Chen, 2011). Extant research identified other antecedents of CGIP, whereas the mechanism remains relatively unexplored. We disclosed the potential mechanism by exploring the mediating effect of GOI, which provides valuable insights into how employees' PEB could increase CGIP.

Third, we considered the moderating effect of LPEB, which is mainly discussed as an antecedent of employees' PEB (Kitchell, 1995). This study shows that the relationship between employees' PEB and LPEB could be more comprehensive. Finally, we also referred to IR and more specifically divided the construct into two dimensions, which contributes to the existing research by extending the different influences of both technology and resource innovation resistance. The relationship between employees' PEB and CGIP is unpredictable in most cases, which could increase uncertainties about the effect of employees' PEB. Moreover, as far as the internal and external factors are concerned, the aforementioned relationship could be more complex and vary from stage to stage (Li et al., 2022). Therefore, companies might endeavor to effectively identify and evaluate different types of internal and external factors that could affect CGIP. This kind of detailed research offers in-depth insights into CGIP.

### Practical implications

This research also offers several practical implications for organizations that wish to improve their environmental responsibility and green innovation performance. First, the company's leader should highlight and encourage the employees' pro-environmental behaviors in the workplace. This study shows that employees' behavior would directly affect the green innovation performance of the company. Since employees' PEB is related to a long-term habit or belief (Afsar and Umrani, 2020), it can be considered in the recruitment process of the company.

Second, since GOI is an important part of the company culture (Kitchell, 1995) and acts as a mediator in the relationship between employees' PEB and CGIP, the company can frame some policies and activities to increase the employees' group identity. In this way, firms need to create a pro-environmental atmosphere or culture and actively promote the green identity of employees so as to improve the performance of CGIP.

Third, leaders can act or present their pro-environmental behaviors in the workplace to help or motivate their employees to increase their awareness toward green innovation. We observed that the leader has a positive mediating effect on the CGIP, which means that both the leader and the employee play an important role in the company's performance. Apart from the factors inside the corporate, innovation resistance would also affect the green innovation performance according to the results of this study. Thus, the company can also follow up on the latest technologies and enrich the resources, which can help improve green innovation performance from a managerial view.

### Limitations and further research

As research focuses on employees' workplace pro-environmental behaviors remain sparse, several areas for future research are offered. First, in addition to the variables discussed in this article, other variables may predict or affect corporate green innovation performance, for example, self-interest and prosocial motives (Bamberg and Möser, 2007). In addition, with the deepening of environmental reform efforts and stricter requirements of environmental policies, the reform of green innovation is gradually affecting the service industry; thus, future research can be conducted in service industries, especially in the emerging markets where the green economy is still immature (Hiz et al., 2018; Stucki et al., 2018). For example, more and more restaurants transform their menu into a digital format and encourage the consumers to order with tablets. Thus, it would be valuable to conduct more future research on the influence of additional variables and to explore more possible mechanisms on the impact of employees' PEB on GIP.

Second, all the previous studies relied on the same source, self-reported data, which raises concerns about the effects of common method variance (Podsakoff et al., 2012). Future studies should use multiple methods to collect data and use a multidimensional view to measure variables, such as second-hand data and bilateral data, which can provide a more comprehensive and objective view to measure and explore the behaviors (Bamberg and Möser, 2007). For example, videos should be collected from the employees' workplace to find out their PEB instead of asking them to fill in the questionnaire. Thus, future studies are encouraged to examine the relationships between employees' PEB and GIP by applying a new method, such as field study, and using new components of the mediators and moderators.

## Conclusion

This study focused on corporate GIP from the employees' perspective, which plays an important role in corporate performance but has not been fully taken into account as a dependent variable, and identified the mediating effect of GOI, as well as the moderating effect of leaders' PEB and innovation resistance. It contributes to the existing literature by considering the consequence of employees' PEB and combined both micro- and macro-level elements of organizational green management in the study framework. This study also provides important theoretical and practical implications. Finally, it points out the limitations of this research and proposes ideas that can be explored in future studies.

## Data availability statement

The raw data supporting the conclusions of this article will be made available by the authors, without undue reservation.

## Author contributions

All authors listed have made a substantial, direct, and intellectual contribution to the work and approved it for publication.

## Funding

XD acknowledges the support of the National Natural Science Foundation of China (No. 72102005). BW acknowledges the support of the National Natural Science Foundation of China (Grant 71902148) and the System Science and Enterprise Development Research Center (Xq22B02).

## Conflict of interest

ZC is employed by Wuliangye Yibin Co., Ltd. The remaining authors declare that the research was conducted in the absence of any commercial or financial relationships that could be construed as a potential conflict of interest.

## Publisher's note

All claims expressed in this article are solely those of the authors and do not necessarily represent those of their affiliated organizations, or those of the publisher, the editors and the reviewers. Any product that may be evaluated in this article, or claim that may be made by its manufacturer, is not guaranteed or endorsed by the publisher.
