# Corrigendum: The impact of employees' pro-environmental behaviors on corporate green innovation performance: The mediating effect of green organizational identity

**DOI:** 10.3389/fpsyg.2023.1130314

**Published:** 2023-02-28

**Authors:** Zujie Cheng, Banggang Wu, Xiaoyu Deng, Wei Li

**Affiliations:** ^1^Wuliangye Yibin Co., Ltd., Yibin, China; ^2^Business School, Sichuan University, Chengdu, China; ^3^Business School, Beijing Technology and Business University, Beijing, China

**Keywords:** green innovation performance, green organizational identity, innovation resistance, employees' pro-environmental behaviors, leader's pro-environmental behaviors

In the published article, an author name was incorrectly written as Zhujie Cheng. The correct spelling is Zujie Cheng.

The authors apologize for this error and state that this does not change the scientific conclusions of the article in any way. The original article has been updated.

